# The Ethylates, or Caustic Alcohols

**Published:** 1894-08-11

**Authors:** Benjamin Ward Richardson


### The Ethylates, or Caustic Alcohols.

By Sir Benjamin Ward Richardson, M.D., F.R.S.

### SODIUM ETHYLATE IN TREATMENT OF NÆVUS.

In my last communication on the ethylates I reported that I had used the ethylate of sodium in 103 cases of nævus occurring in children, and that in 96 there had been complete success. It will be practical, before leaving this ethylate, to present, from experience, some facts bearing a little more upon the mode of application and after management in such cases.

In the first applications, care was taken to apply the ethylate with a glass rod, and a bottle was constructed in which the stopper played the part of the rod, that is to say, the stopper had extended from it a long pointed rod, which went from the bottom of the bottle to the stopper. When the stopper was lifted out, sufficient of the ethylate remained on the point for application over the surface of the nævus. The object of the glass stopper was to give a point that would not corrode in the presence of the ethylate, and to this day the fluid is often sent out with the stopper and the rod after the original form. In course of time this plan was modified and a camel's hair pencil was used in place of the rod, a new pencil being selected each time if necessary. The plan is not inconvenient except for the expense of the pencil, but now I have a better method still. I take a new quill pen, dip it, as if I were dipping it into ink, into the ethylate and apply the fluid with the point of the pen. The quill resists the action of the ethylate very fairly, and when the nib is destroyed I cut it off and make a new nib for the purpose, so that one quill will last ten or twelve times.

The nævus is first dried with lint or a soft towel and then the surface is coated over with the ethylate; there is no fear of the fluid running over the edge of the nævus as there is with nitric acid, for, to use a common term, the liquid "bites" at once, and does not leave the spot to which it is applied. It causes a little stinging sensation; in a short time the skin looks in a redder condition than it was before, and from it there oozes minute points of a clear serous or faintly red fluid; then the skin dries, and a thin but firm scale or scab forms, and remains for two or three days, when it begins to exfoliate. In cases where the nævus is well raised from the surface—the common form of vascular raised nævus—it sometimes happens that when the scab exfoliates the surface underneath is found to present a delicate but otherwise normal skin, the cure being effected by one application, but as this does not occur in more than two or three per cent. of instances it need not be looked for as a likely event. In the ordinary case when the scale begins to exfoliate it will be found that there is still a raised red surface, in fact, an undestroyed portion of the vascular layer, indicating that the ethylate must be again applied. In order to reapply I was accustomed, originally, to raise the scale from the skin by means of a fine wire scoop, and when the exposed surface was quite cleared to paint it over again with the solution. The operation is effective but painful, and from its being attended with pain I was led to modify the process as follows. I take a slightly curved needle, with a cutting shoulder, shaped like one of the old couching needles, and with this needle I make three or four punctures through the scab or scale into the nævus. If a slight escape of blood is caused I take the blood up with a fold of lint or cotton wool, and then lay on a layer of the ethylate solution over the scale, allowing the fluid to run into the openings. Very little pain is experienced, and the new destruction is sufficiently active. Then after three days should there be still a boggy feeling under the scale I repeat the process with the needle, and when the scale begins to come off, leaving the sense of a firm flat surface beneath, the exfoliation is permitted to go on until the whole is removed, when in the usual course the skin is found and left natural. The needle has been referred to as of value for this second or third application, but if a needle be not at hand a pointed quill, used in the manner already directed, can be made to take its place.

For the treatment of the ordinary raised nævus the ethylate treatment may be considered as near to a specific remedy as ever can be expected, and I have wondered, sometimes, why such a certain, safe and simple means of cure is not placed on the canons of treatment as true and sufficient for the purpose required. It has several times occurred to have brought or sent to me, cases in which other means have been attempted, as the cautery, the ligature, the electric point, vaccination, the application of nitric acid, or excision, none of which have been successful; but ethylate has been quite certain and curative in its usefulness. Nor does the size or position of the raised nævus materially modify the result. A child seven years of age was under my care in whom a large raised nævus was situated on the left eyelid. The growth included the whole of the upper outer surface of the lid and extended to the forehead in a semi-circle a quartei of an inch in width in its widest part. It was a serious deformity, and owing to its proximity to the eyeball promised to be serious to treat. There was, however, no difficulty; all that was wanted was a little extra delicacy of manipulation and a few more applications than usual. In the end I never had a better termination of a case; two years after the operation it would have been impossible to have told that the disfigurement had ever existed; there was no trace of a scar, and, indeed, I have never seen a scar from the treatment; the natural state of the skin that follows is the most satisfactory part of the treatment when treatment ends in cure.

The application is very successful in case of warts, moles, and hirsute growths. A young lady who had a large raised brown coloured mole on the right cheek, and who had been advised that, although it could be easily removed by a slight and perfectly safe operation by the knife, a scar would be left, asked me, on the suggestion of her medical attendant, whether it could be removed by the ethylate without the production of a scar. My answer was that it could be, and the treatment came under my hands. The excrescence had lasted for many years and was a considerable annoyance. It was easily exfoliated by the application of the ethylate, the destroyed portions coming off in small dead segments, and when the level surface of the skin was reached and the last scale was raised it was impossible to detect the faintest scar or roughness of surface.

